# Antibacterial Properties of Organosulfur Compounds of Garlic (*Allium sativum*)

**DOI:** 10.3389/fmicb.2021.613077

**Published:** 2021-07-27

**Authors:** Sushma Bagde Bhatwalkar, Rajesh Mondal, Suresh Babu Naidu Krishna, Jamila Khatoon Adam, Patrick Govender, Rajaneesh Anupam

**Affiliations:** ^1^Department of Biotechnology, Dr. Harisingh Gour Vishwavidyalaya (A Central University), Sagar, India; ^2^Indian Council of Medical Research, Bhopal Memorial Hospital & Research Centre, Bhopal, India; ^3^Department of Biomedical and Clinical Technology, Durban University of Technology, Durban, South Africa; ^4^School of Life Sciences, University of KwaZulu-Natal, Durban, South Africa

**Keywords:** garlic (*A. sativum*), organosulfur compounds, antibiofilm, antibacterial, multi-drug resistance (MDR)

## Abstract

Garlic (*Allium sativum*), a popular food spice and flavoring agent, has also been used traditionally to treat various ailments especially bacterial infections for centuries in various cultures around the world. The principal phytochemicals that exhibit antibacterial activity are oil-soluble organosulfur compounds that include allicin, ajoenes, and allyl sulfides. The organosulfur compounds of garlic exhibit a range of antibacterial properties such as bactericidal, antibiofilm, antitoxin, and anti-quorum sensing activity against a wide range of bacteria including multi-drug resistant (MDR) strains. The reactive organosulfur compounds form disulfide bonds with free sulfhydryl groups of enzymes and compromise the integrity of the bacterial membrane. The World Health Organization (WHO) has recognized the development of antibiotic resistance as a global health concern and emphasizes antibiotic stewardship along with the urgent need to develop novel antibiotics. Multiple antibacterial effects of organosulfur compounds provide an excellent framework to develop them into novel antibiotics. The review provides a focused and comprehensive portrait of the status of garlic and its compounds as antibacterial agents. In addition, the emerging role of new technologies to harness the potential of garlic as a novel antibacterial agent is discussed.

## Introduction

Garlic (*Allium sativum*), belonging to family *Liliaceae*, mainly the bulb of garlic, has been used as a spice in cooking worldwide especially in Italy and Southeast Asia. More importantly, garlic has been an ingredient in folk and traditional medicine since ancient times (Rivlin, 2001). Garlic is cultivated all over the world with a per-capita consumption of two pounds per year. As per the Food and Agricultural Organization of the United Nations, China and India are first and second, respectively, in average (1961–2017) garlic production. Health benefits that are associated with the use of garlic are attributed to its anticancer, anti-inflammatory, antifungal, antiviral, and antibacterial activity. Several *in vitro*, *in vivo*, and epidemiological studies indicate that garlic exhibits anticancer activity, and the likely mechanism of action is by activating metabolizing enzymes, inhibiting reactive oxygen species, radical scavenging, preventing DNA damage, and tumor inhibition (Cao et al., 2014; Zhang Y. et al., 2019). The immunomodulatory effects of garlic are mediated through its ability to modulate cytokine production as well as activate immune response by stimulating antibody secretion and immune cells (Arreola et al., 2015). Garlic displays anti allergic properties by inhibiting antibody-mediated histamine production and modulates airway allergic response (Kyo et al., 2001; Zare et al., 2008). The anti inflammatory and anti arthritic ability of garlic comes from its ability to inhibit NF-κB signaling (Ban et al., 2009). Garlic oil (GO) exhibits antifungal activity against *Candida albicans* and *Penicillium funiculosum* by penetration into cells and organelles and causing differential expression of genes that are critical for cellular metabolism (Li et al., 2016). One of the earliest reports of garlic’s antibacterial activity was by Small et al. (1947) and Stoll and Seebeck (1947). Since then, extensive research has been performed on the antibacterial effects of garlic. The antibacterial activity against various pathogenic and drug-resistant bacteria was tested using crude garlic extracts, garlic powder (GP), garlic extracts using various solvents, GO, and phytochemicals isolated from garlic. The constant and rapid emergence of antimicrobial resistance has been recognized as an alarming threat to human health, which mandates the scientific community to develop novel and effective antibacterial agents (Cheng et al., 2016). Garlic compounds exhibit multiple modes of antibacterial activity and have enormous potential to be developed into novel antibacterial agents. Most reviews about garlic discuss the antibacterial activity of garlic as one of its many health benefits diluting the importance of garlic compounds as potential antibacterial agents. This review exclusively focuses on significant antibacterial studies that were performed with garlic and its phytochemicals.

## Active Phytochemicals of Garlic

**FIGURE 1:**
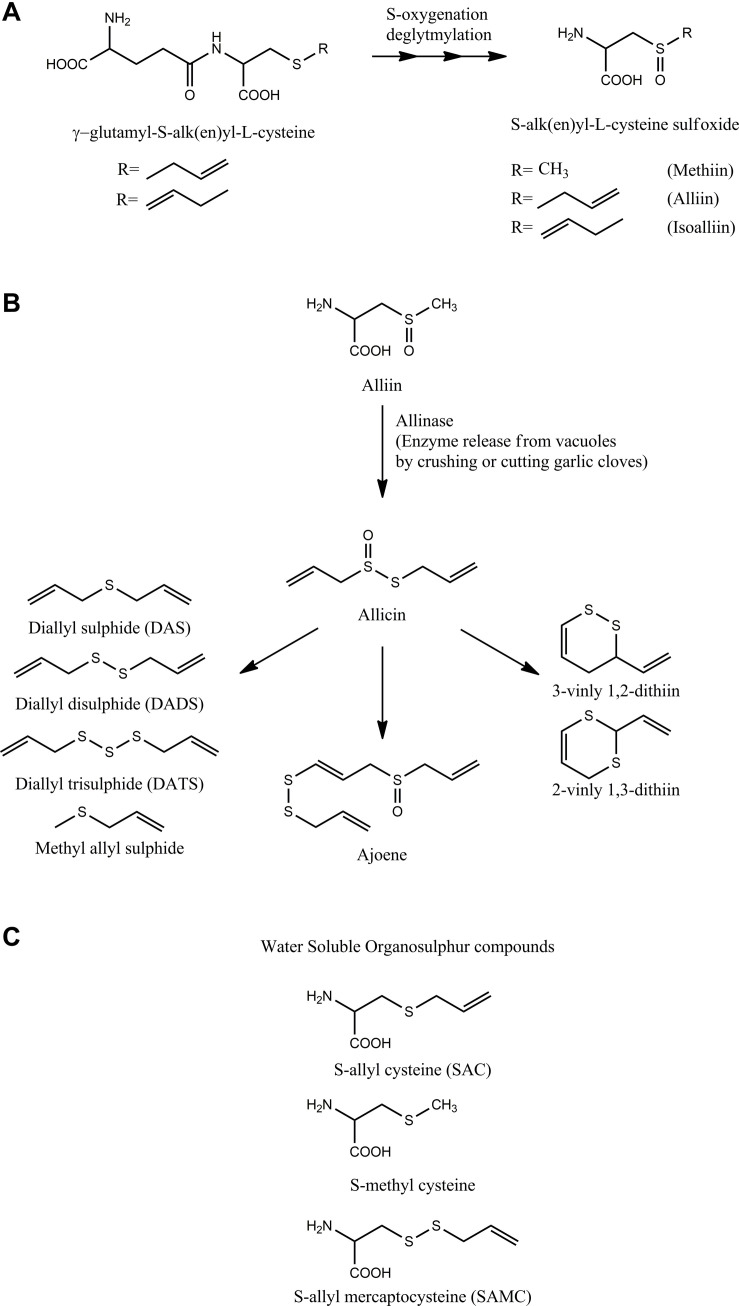
Organosulfur compounds of garlic: The figure shows the major organosulfur compounds present in garlic. **(A)** The major compounds found in intact garlic cloves. **(B)** The crushing of garlic clove converts alliin into allicin by the action of allinase enzyme. Allicin is a highly unstable compound that degrades and rearranges itself into different organosulfide compounds shown in the figure. **(C)** Apart from oil-soluble organosulfur compounds, garlic also has water-soluble organosulfur compounds shown in the figure.

Most of the health benefits of garlic are attributed to a myriad of cysteine-derived sulfur-containing organic compounds present in garlic (extensively reviewed in Fenwick and Hanley, 1985a,b,c). The organosulfur compounds of intact garlic clove greatly differ from that present in garlic juice obtained after crushing garlic. The intact garlic mainly contains non-volatile γ-glutamyl-*S*-alk(en)yl-L-cysteines, namely, γ-glutamyl-*S*-allyl-L-cysteine, γ-glutamyl-*S-trans*-1-propenyl-L-cysteine, and *S*-alk(en)yl-L-cysteine sulfoxides such as *S*-allyl-L-cysteine sulfoxide (alliin), *S*-(*trans*-1-propenyl)- L-cysteine sulfoxide (isoalliin), and *S*-methyl-L- cysteine sulfoxide (methiin) with a small amount of *S*-allyl cysteine (SAC) (Figure 1A) (Block, 1992). Crushing or cutting garlic cloves releases allinase enzyme sequestered in the vacuoles, which encounters cytosolic alliin to convert it into an array of thoisulfinates of which the most prominent is allicin. The highly reactive, unstable, and volatile allicin decomposes to yield a large number of sulfides such as diallyl sulfide (DAS), diallyl disulfide (DADS), diallyl trisulfide (DATS), methyl allyl disulfide (MADS), methyl allyl sulfide, ajoene, and vinyl dithiins (2-vinyl-1,3-dithiin, 3-vinyl-1,2-dithiin) shown in Figure 1B (Brodnitz et al., 1971). The sulfides are oil-soluble compounds that are responsible for the characteristic garlic odor and flavor. Allicin exhibits excellent *in vitro* antibacterial activity, which resulted in a huge number of studies to evaluate the potential of allicin and oil-soluble organosulfur compounds of garlic as antibacterial agents (Cavallito and Bailey, 1944a). A large body of literature supports the antibacterial potential of garlic organosulfides. The organosulfur compounds present in the aqueous and alcoholic extract of garlic include *S*-allyl cysteine (SAC), *S*-allylmercapto-L-cysteine (SAMC), and *S*-methyl cysteine (Figure 1C). The compounds are non-volatile, non-odiferous, and stable compounds compared to volatile organosulfides. Most health benefits of garlic are largely attributed to these organosulfur compounds present in garlic. However, garlic organosulfur compounds are very unstable with low bioavailability and the presence of these compounds depends on the processing of the garlic during the preparation of garlic supplements (Amagase et al., 2001; Amagase, 2006).

The main antibacterial organosulfur compounds of garlic are allicin, ajoene, and various aliphatic sulfides. The extraction procedure results in concentrating a particular compound rather than providing a pure compound. Extraction of garlic with water or ethanol and concentrating the extract will provide an allicin-rich product. It was noticed that yield with ethanol is better compared to water (Fujisawa et al., 2008). However, extraction of concentrated ethanolic distillate with organic solvent yields a highly concentrated and pure allicin product (Cavallito and Bailey, 1944a; Ratnakar and Murthy, 1995). Later, it was reported that extraction using acetone will yield higher allicin compared to ethanol (Canizares et al., 2004a). Recently, salting-out extraction using ethanol and ammonium sulfate result in effective extraction of allicin (Li et al., 2017). Oil-macerated garlic extract has a very high proportion of ajoene along with other thiosulfinates (Yoshida et al., 1998). Steam distillation of garlic yields GO, which mainly consists of various aliphatic sulfides (Avato et al., 2000). The components of both oil-macerated garlic extract and GO can be separated using chromatographic and distillation techniques (Casella et al., 2013).

PubMed search of “Garlic antibacterial” yields more than 350 research papers. This large body of literature comprises research papers that investigated the antibacterial activity of crude preparation of garlic, various extracts of garlic, and individual organosulfur compounds of garlic against various bacteria including MDR bacteria. Table 1 provides a list of *in vitro* antibacterial activity of various garlic products and compounds against different bacteria. Similarly, Table 2 provides similar information on *in vivo* studies. Some of the early research that reported the antibacterial activity of garlic against a wide variety of bacteria has been summarized by Adetumbi and Lau (1983). The present review provides a comprehensive summary of this large body of research.

**TABLE 1:** *In vitro* antibacterial activity of various garlic products and compounds.

Source	Bacteria	References
Crude or fresh garlic extract	*S. aureus E. coli S. typhi L. monocytogenes* MDR STEC *C. jejuni Vibrio parahaemolyticus Mycobacterium* species MRSA *Bacillus subtilis S. mutans C. difficile C. perfringens Bacteroides* species *Lactobacillus casei* MDR *P. aeruginosa* MDR *K. pneumoniae* MDR *Serratia marcescens*	Kumar and Berwal, 1998; Vuddhakul et al., 2007; Dakka, 2011; Lu et al., 2011a; Viswanathan et al., 2014; Jain et al., 2015b; Roshan et al., 2017; Farrag et al., 2019
Garlic powder	*S. typhimurium E. coli H. pylori B. cereus E. coli* (O55, O128, and O112) *Shigella* species *Vibrio* species *Yersinia enterocolitica L. monocytogenes S. enterica Campylobacter* species *Bacteroides fragilis B. subtilis Enterobacter aerogenes Enterococcus faecalis Klebsiella aerogenes Proteus vulgaris Lactobacillus acidophilus Streptococcus faecalis S. mutans Streptococcus pyogenes*	Johnson and Vaughn, 1969; O’Gara et al., 2000; Ross et al., 2001
Garlic paste	*E. coli* O157:H7	Gupta and Ravishankar, 2005
Aqueous garlic extract	*B. cereus* MDR *Shigella* species MDR *E. coli H. pylori S. aureus Bacillus sphaericus S. epidermidis E. aerogenes P. aeruginosa S. typhi S. pneumoniae K. pneumoniae Streptococcus pyogenes Sh*. species *E. coli Proteus* species *H. influenzae S. mutans* MDR *S. mutans Streptococcus* species *Actinomyces naeslundii E. faecalis*	Saleem and Al-Delaimy, 1982; Chowdhury et al., 1991; Cellini et al., 1996; Sivam et al., 1997; Arora and Kaur, 1999; Dikasso et al., 2002; Iwalokun et al., 2004; Bakri and Douglas, 2005; Ruddock et al., 2005; Fani et al., 2007; Gupta et al., 2010; Gull et al., 2012; Meriga et al., 2012; Velliyagounder et al., 2012; Mozaffari Nejad et al., 2014; Wallock-Richards et al., 2014; Pavlovic et al., 2017; Jang et al., 2018
	*Actinobacillus actinomycetemcomitans Prevotella intermedia Prevotella nigrescens Porphyromonas gingivalis, Fusobacterium nucleatum Leptotrichia buccalis N. gonorrhoeae* MDR *M. tuberculosis M. tuberculosis B. subtilis Burkholderia cepacia* complex *Proteus mirabilis Salmonella enteritidis*	
Ethanolic garlic extract	*H. pylori M. tuberculosis* MDR *M. tuberculosis E. coli Enterobacter species P. aeruginosa Proteus species Klebsiella species S. aureus Bacillus species* VRSA *S. pneumoniae B. cereus K. pneumoniae S. mutans Proteus mirabilis Salmonella enteritidis E. aerogenes E. faecalis Lactobacillus paracasei Lactobacillus rhamnosus MRSA S. epidermidis Streptococcus oralis Streptococcus sanguis Streptococcus sobrinos Eikenella corrodens*	Hannan et al., 2011; Karuppiah and Rajaram, 2012; Snowden et al., 2014; Jain et al., 2015a; Liaqat et al., 2015; Mohsenipour and Hassanshahian, 2015; Pavlovic et al., 2017; Vlachojannis et al., 2018
Methanolic garlic extract	*E. coli P. aeruginosa S. aureus E. aerogenes E. faecalis Proteus mirabilis Salmonella enteritidis*	Pavlovic et al., 2017
Chloroform garlic extract	*B. cereus S. mutans*	Jain et al., 2015a; Jang et al., 2018
Hexane garlic waste extract	*S. aureus* MRSA	Nakamoto et al., 2020
Garlic oil	*M. tuberculosis H. pylori S. aureus* MRSA *B. cereus E. coli* (O55, O128, and O112) *Shigella* species *Vibrio* species *Yersinia enterocolitica L. monocytogenes*	Jain, 1998; Avato et al., 2000; O’Gara et al., 2000; Ross et al., 2001; Tsao S. and Yin, 2001; Tsao S.M. and Yin, 2001; Kim et al., 2004; Casella et al., 2013; Robyn et al., 2013; Mnayer et al., 2014; Viswanathan et al., 2014
	*S. enterica Campylobacter* species *Bacteroides fragilis* *E. aerogenes* *E. faecalis* *K. aerogenes* *P. vulgaris* *L. acidophilus* *S. faecalis* *S. mutans* *S. pyogenes* *K. pneumoniae* *P. aeruginosa* MDR *K. pneumoniae* MDR *P. aeruginosa* *S. typhi* *C. jejuni*	
Ajoene	*B. cereus* *B. subtilis* *S. aureus* *Mycobacterium smegmatis* *Mycobacterium phlei* *M. tuberculosis* *M. luteus* *L. plantarum* Streptococcus species *Streptomyces griseus* *E. coli* *K. pneumoniae* *P. Aeruginosa* *X. maltophilia* *Cronobacter sakazakii*	Naganawa et al., 1996; Yoshida et al., 1998; Feng et al., 2014; Viswanathan et al., 2014
Z-10-devinylajoene and iso-E-10-devinylajoene	*B. cereus* *B. subtilis* *S. aureus* *M. phlei* *M. luteus* *E. coli* *K. pneumoniae* *P. aeruginosa* *X. maltophilia*	Yoshida et al., 1998, 1999
Diallyl sulfide (DAS)	*H. pylori* *S. aureus* MRSA *K. pneumoniae* *P. aeruginosa* *S. typhimurium* *E. coli* O157:H7 *L. monocytogenes* *Staphylococcus aureus* *C. jejuni* *A. actinomycetemcomitans* *Cronobacter sakazakii*	O’Gara et al., 2000; Tsao S. and Yin, 2001; Tsao S.M. and Yin, 2001; Yin and Cheng, 2003; Lu et al., 2011a, b; Velliyagounder et al., 2012; Feng et al., 2014
Diallyl disulfide (DADS)	*H. pylori* Clarithromycin-resistant *H. pylori* Metronidazole-resistant *H. pylori* *S. aureus* MRSA *K. pneumoniae* *P. aeruginosa*	O’Gara et al., 2000; Tsao S. and Yin, 2001; Tsao S.M. and Yin, 2001; Yin and Cheng, 2003; Liu et al., 2008; Lu et al., 2011a; Casella et al., 2013; Robyn et al., 2013
	*S. typhimurium E. coli E. coli* O157:H7 *L. monocytogenes Staphylococcus aureus C. jejuni*	
Diallyl trisulfide (DATS)	*H. pylori* Clarithromycin-resistant *H. pylori* Metronidazole-resistant *H. pylori S. aureus* MRSA *K. pneumoniae P. aeruginosa Leuconostoc mesenteroides Pediococcus pentosaceus Lactobacillus plantarum C. jejuni*	O’Gara et al., 2000; Tsao S. and Yin, 2001; Tsao S.M. and Yin, 2001; Kim et al., 2004; Liu et al., 2008; Lu et al., 2011a
Diallyl tetrasulfide (DATTS)	*H. pylori S. aureus* MRSA *K. pneumoniae P. aeruginosa*	O’Gara et al., 2000; Tsao S. and Yin, 2001; Tsao S.M. and Yin, 2001
Mixture of diallyl sulfides (DASS)	*E. aerogenes E. coli S. enterica S. sonnei L. monocytogenes Y. enterocolitica M. tuberculosis*	Ross et al., 2001; Oosthuizen et al., 2017
Dimethyl trisulfide	*E. aerogenes E. coli S. enterica S. sonnei L. monocytogenes Y. enterocolitica S. aureus L. mesenteroides P. pentosaceus L. plantarum*	Ross et al., 2001; Kim et al., 2004
Ally methyl sulfide (AMS)	*Actinobacillus pleuropneumoniae*	Becker et al., 2012
Allicin	*S. aureus* MRSA *Streptococcus* species *Bacillus* species *V. cholerae M. tuberculosis Mycobacterium* species *Enterococci* species *H. pylori S. epidermidis* Methicillin-resistant *S. epidermidis* Lancefield group B *streptococci E. coli A. actinomycetemcomitans C. jejuni* Bcc *C. difficile P. aeruginosa S. pyogenes*	Cavallito and Bailey, 1944a; Rao et al., 1946a, b; Delaha and Garagusi, 1985; Jonkers et al., 1999a; O’Gara et al., 2000; Perez-Giraldo et al., 2003; Canizares et al., 2004b; Cutler and Wilson, 2004; Cutler et al., 2009; Fujisawa et al., 2009; Velliyagounder et al., 2012; Robyn et al., 2013; Wallock-Richards et al., 2014; Roshan et al., 2017, 2018; Fuchs et al., 2018

**TABLE 2:** *In vivo* antibacterial activity of various garlic products and compounds.

Source	Bacteria	Animal model	References
Aqueous garlic extract	*Shigella flexneri Y*	Rabbit	Chowdhury et al., 1991
Aqueous extract of toluene garlic extract	*P. aeruginosa*	*Caenorhabditis Elegans*	Rasmussen et al., 2005
		Mice	Bjarnsholt et al., 2005
Ajoene	*P. aeruginosa*	Mice	Jakobsen et al., 2012
DAS	MRSA	Mice	Tsao et al., 2007
DADS	MRSA	Mice	Tsao et al., 2007
Ally methyl sulfide (AMS)	*Actinobacillus pleuropneumoniae*	Pig	Becker et al., 2012
Allicin	*H. pylori Aeromonas hydrophila*	Meta-analysis of clinical studies, rainbow trout (fish)	Nya et al., 2010; Si et al., 2019*

*Clinical study.

## Antibacterial Activity of Garlic Fresh Extract and Powder

Garlic is one of the popular spices added to food to enhance the flavor, and it has been used in different cultures and traditions around the world to treat bacterial infections for centuries. Several studies have evaluated the antibacterial activity of various garlic preparations such as crude or fresh garlic extract (FGE), and garlic paste. The antibacterial activity of garlic paste and FGE against commensal and pathogen enteric bacteria such as *Escherichia coli, E. coli* O157:H7, *Salmonella* species, *Shigella* species, *Vibrio* species, *Campylobacter* species, *Listeria monocytogenes*, *Enterobacter*, and *Enterococcus* species, *Lactobacillus acidophilus*, *Staphylococcus aureus, Streptococcus* species, and *Clostridium difficile* has been reported by various laboratories (Johnson and Vaughn, 1969; Kumar and Berwal, 1998; Ross et al., 2001; Gupta and Ravishankar, 2005; Vuddhakul et al., 2007; Lu et al., 2011b; Jain et al., 2015a; Roshan et al., 2017). These studies suggest that garlic consumption could help in preventing food poisoning. In addition, various studies have evaluated the impact of garlic and its organosulfur compounds on the gut microbiome. Garlic was found to positively influence the gut microbiome and protect the gut microbiome damage from high-fat diet (Chen et al., 2019). Supplementing feed of farrowing sows and European bass with GO decreased pathogenic microbes from the gut microbiome (Rimoldi et al., 2020; Satora et al., 2020). Allicin treatment prevented high carnitine diet-induced dysbiosis to lower the atherosclerosis risk factor trimethylamine N-oxide that is produced by the gut microbiome (Wu W. K. et al., 2015). Oral administration of alliin, precursor of allicin, to rats resulted in decreasing the relative abundance of only *Allobaculum* genus in the cecum (Zhang C. et al., 2019). The gut microbiome was altered upon intragastric administration of DADS of rat, a low dose of DADS decreased *Bacteroidetes* phyla but increased *Firmicutes* phyla bacteria (Yang et al., 2019). Oral administration of propyl propane thiosulfonate restored the richness and evenness of gut microbiome lost due to dextran sodium sulfate-induced colitis in mice (Vezza et al., 2019). In a small-scale clinical trial, aged garlic extract supplementation for 3 months increases the richness and diversity of the gut microbiome with increase in *Lactobacillus* and *Clostridium* species (Ried et al., 2018). All the studies indicate that garlic and its compounds have a positive effect on gut microbiome composition and richness. However, the mechanistic details still need to be investigated. In a recent study from our laboratory, FGE exhibited activity against MDR Shiga-toxin producing *E. coli* (STEC) isolates from clinical and food samples (Bhatwalkar et al., 2019). In addition to antibacterial activity, garlic crude and aqueous extract exhibited anti-adherent activity against the standard strain type of *Streptococcus mutans* (Jain et al., 2015a). The data suggest that garlic could be used to preserve food and prevent foodborne infections. However, the antibacterial activity was dramatically decreased when experiments were performed with buffered peptone water and ground beef, suggesting that further research is required to utilize garlic as a food/meat-preserving agent (Gupta and Ravishankar, 2005). The causative agent of gastric ulcers, *Helicobacter pylori* (standard strains types and clinical isolates), was found to be sensitive to GP and 1,000 μg/ml of GP inactivated *H. pylori* at 6 h in a time course viability assay (O’Gara et al., 2000). Allicin-rich crude extract exhibited better antibacterial activity against *Mycobacterium phlei, Mycobacterium smegmatis*, and *Mycobacterium tuberculosis* compared to isoniazid and ethambutol. Also, disk diffusion assay with allicin-rich extract exhibited significant activity against MRSA (Viswanathan et al., 2014). Another study also found that FGE was effective against MDR strains of *E. coli, Pseudomonas aeruginosa, Klebsiella pneumoniae, Serratia marcescens*, and MRSA in both *in vitro* and *in vivo* assays (Farrag et al., 2019).

## Antibacterial Activity of Garlic Aqueous Extract

There are several reports of antibacterial activity of aqueous garlic extract (AGE) against a variety of bacteria. *In vitro* assay with AGE (10%) showed complete inhibition of *Bacillus cereus* and the activity varies upon the storage conditions and heat treatment of the aqueous extract (Saleem and Al-Delaimy, 1982). AGE exhibited *in vitro* antibacterial activity against various pathogenic bacteria including *Shigella* and *Salmonella* species and enterotoxigenic *E. coli* (Arora and Kaur, 1999). In addition, AGE fully cured the rabbits that were challenged with *Sh. flexneri Y* by completely clearing them of bacteria with no significant side effects (Chowdhury et al., 1991).

Supporting the results obtained with GP, *in vitro* assays indicated that *H. pylori* is sensitive to AGE, and the sensitivity was more compared to *S. aureus* (Cellini et al., 1996; Sivam, 2001). *In vitro* antibacterial assays report that AGE is effective against various Gram-positive and Gram-negative oral bacteria, which include periodontal pathogenic bacteria *Porphyromonas gingivalis, Aggregatibacter actinomycetemcomitans*, and *S. mutans* (Bakri and Douglas, 2005; Fani et al., 2007; Velliyagounder et al., 2012). Different studies reported that AGE exhibited activity against a large variety of Gram-positive and Gram-positive pathogenic bacteria including MDR strains and isolates such as MDR *M. tuberculosis* showing not only the effectiveness of garlic against drug-resistant bacteria but also its broad spectrum (Iwalokun et al., 2004; Gupta et al., 2010; Gull et al., 2012; Meriga et al., 2012). In an interesting study, it was found that counts of *S. aureus* in hamburger upon addition of AGE reduced in a dose-dependent manner during storage for different time points in the fridge and freezer, supporting the idea of using garlic for meat preservation (Mozaffari Nejad et al., 2014). To compare the antibacterial activity of various garlic health products, aqueous extracts of different products that included GP, GO, gelatinous GP suspension, aged garlic extract, and gelatinous suspension of aged garlic extract were prepared along with fresh garlic. All the extracts exhibited activity against *Neisseria gonorrhoeae, S. aureus*, and *Enterococcus faecalis*. The activity was correlated to the amount of fresh garlic constituents, namely, allicin and SAC, present in the products (Ruddock et al., 2005). The *Burkholderia cepacia* complex (Bcc) consists of 17 different species of soil bacteria that are pathogenic to allium species. These bacteria cause life-threatening lung infections in patients suffering from cystic fibrosis. AGE exhibited activity against Bcc, and this activity correlated with the allicin content of the extract (Wallock-Richards et al., 2014). In a recent study, non-aged and aged garlic cloves were pressed to remove their juices, dried, and powdered before extracted with water, ethanol, and chloroform. All three extracts of aged garlic exhibited antibacterial activity while only chloroform extract of non-aged garlic had activity against *B. cereus* (Jang et al., 2018). All these studies indicate that allicin is the main phytochemical responsible for the antibacterial activity of AGE. Although the ethanol extract of garlic also has allicin, AGE is more effective due to the presence of other antibacterial chemicals, which might result in a synergistic or additive effect.

## Antibacterial Activity of Garlic Ethanolic Extract

HPLC analysis of ethanolic extract of garlic (EGE) revealed that it contains various thoisulfinates, the major one being allicin. The anti-*H. pylori* activity of this extract decreased with the decrease in the concentration of allicin. Furthermore, it was seen that the maturation of garlic increases the allicin yield and extract with acetone yielding a higher percentage of allicin compared to ethanol (Canizares et al., 2004a, b). *In vitro* studies have reported that EGE was found to show antibacterial activity against various pathogenic bacteria including MDR bacteria, MDR *M. tuberculosis* isolates, and vancomycin-resistant *S. aureus* (VRSA) isolates (Hannan et al., 2011; Karuppiah and Rajaram, 2012; Snowden et al., 2014; Liaqat et al., 2015). The antibacterial and antiadherence activity of organic solvent (chloroform, acetone, and ethanol) extracts of garlic was least compared to crude and aqueous extract against *S. mutans* (Jain et al., 2015a). The leaves of wild garlic (*Allium ursinum* subsp. *ucrainicum*) found in Serbia were extracted with 70 and 96% ethanol and 80% and absolute methanol, and the *S*-alk(en)ylcysteines (alliin, isoalliin, and methiin) content of the extracts was analyzed using NMR studies. The extracts exhibited some degree of antibacterial activity against test enteropathogenic bacterial strains with *Salmonella enteritidis* being the most sensitive. The tested bacteria were more sensitive to ethanolic extract compared to other extracts (Pavlovic et al., 2017). However, the study should have determined the amount of allicin in the extracts for better interpretation of the results instead of alliin, which is a precursor of allicin. Ethanolic (30%) extract of fermented black garlic exhibited antibacterial activity against 11 bacterial strains that cause oral diseases. Short and long incubation with this extract inhibited the growth of more than 90% of salivary bacteria (Vlachojannis et al., 2018). Water extract of the Toluene extract of garlic has been reported to decrease the mortality of *Caenorhabditis elegans* from *P. aeruginosa* infections (Rasmussen et al., 2005) and clear the lungs of mice of *P. aeruginosa* by modulating inflammation (Bjarnsholt et al., 2005). Allicin along with other thoisulfinates present in EGE seems to be responsible for its antibacterial activity. Other than ethanol and methanol extract, the chloroform extract of both aged and non-aged garlic exhibited activity against *B. cereus* by disk diffusion assay (Jang et al., 2018). The hexane extract of solid waste of the GO extraction process exhibited activity against various bacteria including *S. aureus* and MRSA. DASs present in the extract were responsible for this activity (Nakamoto et al., 2020).

## Antibacterial Activity of Garlic Oil

Garlic oil is obtained by steam distillation of macerated or mashed garlic. Reverse-phase high-performance liquid chromatography (HPLC) studies have determined that the GO consists of a large variety of diallyl sulphides and other sulfides (O’Gara et al., 2000; Kim et al., 2004). A recent study has performed an exhaustive analysis of the content of GO and reported that the majority of GO is composed of diallyl and allyl methyl sulfides (Mnayer et al., 2014). The anti-mycobacterium effect of GO was demonstrated using *in vitro* and *in vivo* studies (Jain, 1998; Viswanathan et al., 2014). The anti-*H. pylori* effect of GO was many folds greater than that of GP. This could be because allicin is the only antibacterial thiosulfinate found in GP whereas GO has many organosulfides. The time course viability studies showed concentration-dependent inhibition of *H. pylori* by GO with 64 μg/ml resulting in complete inhibition in 4.5 h (O’Gara et al., 2000). However, two independent clinical studies indicated that administration of garlic GO was unable to ameliorate the *H. pylori* infection (Graham et al., 1999; Aydin et al., 2000). GO has been reported to exhibit antibacterial activity against 14 enteric pathogens and 11 commensal enteric bacteria with commensal bacteria being more sensitive. In time course viability studies, the inhibition of *Enterobacter aerogenes* growth increased with an increase in the concentration of GO, and complete killing was noticed at 22 mg/ml in 1 h (Ross et al., 2001). In another study, different GOs with varying percentages of DDS and DTS along with pure DDS were tested against Gram-positive (*S. aureus* and *Bacillus subtilis*) and Gram-negative (*E. coli* and *P. aeruginosa*). The antibacterial activity was not significant; however, the little activity that was exhibited was found with GO with a higher percentage of DDS. Interestingly, pure DDS showed little activity against only selected tested bacteria (Avato et al., 2000). However, disk diffusion assay found GO to be effective against *S. aureus, E. coli*, *P. aeruginosa*, *B. subtilis*, and MRSA (Casella et al., 2013; Viswanathan et al., 2014). An *in vitro* study tested the activity of GO against 40 *S. aureus* and 60 MRSA isolates and found that GO was more effective against *S. aureus* compared to MRSA, although this activity was significantly less than standard antibiotics (Tsao S.M. and Yin, 2001). Another study by the same group has reported that GO is effective against 237 clinical isolates of *P. aeruginosa* and *K. pneumoniae*, which also included drug-resistant strains. The minimum inhibitory concentration (MIC) values for *P. aeruginosa* were smaller compared to *K. pneumoniae*, and four times MIC of GO eliminated *P. aeruginosa* and *K. pneumoniae* in 16 and 24 h, respectively, in kill curve assays (Tsao S. and Yin, 2001). However, weak antibacterial activity of GO against six different bacteria has been reported using *in vitro* assays (Kim et al., 2004). Other bacteria that were reported to be sensitive to GO are *Salmonella typhi, L. monocytogenes*, and *Campylobacter jejuni* (Robyn et al., 2013; Mnayer et al., 2014). The discrepancy in the antibacterial activity of GO among various *in vitro* studies could be due to the solubility and volatile nature of GO.

## Antibacterial Activity of Ajoene

Allicin can react with itself to yield ajoene, which is found abundantly in oil-macerated garlic. Besides, two ajoene-related compounds Z-10-devinylajoene and iso-E-10-devinylajoene were also isolated from oil-macerated garlic extract. Studies from the Fujino group reported that ajoene and its related compounds were found to display antibacterial activity against several Gram-positive and Gram-negative bacteria including *Mycobacterium* species (Naganawa et al., 1996; Yoshida et al., 1998, 1999). In all these studies, it was noticed that these compounds were more active against Gram-positive bacteria compared to Gram-negative. The same group also reported the antibacterial activity of ajoene and its related compounds against *H. pylori* (Ohta et al., 1999). Mice challenged with *P. aeruginosa* cleared the infection rapidly when treated with ajoene compared to the control group (Jakobsen et al., 2012). Pure ajoene exhibited antibacterial activity against *Cronobacter sakazakii* in a concentration-, time-, and temperature-dependent manner (Feng et al., 2014). More studies testing the activity of ajoene against more bacteria, especially clinical isolates and MDR strains, along with stability and pharmacokinetic studies are needed to better understand and utilize ajoene and its related compounds as antibacterial agents.

## Antibacterial Activity of Garlic Organosulfides

The major constituents of GO are various aliphatic disulfides. DADS, which is the most abundant allyl sulfides in GO. DAS exhibits poor anti-*H. pylori* effect, but this activity increased as the number of sulfurs increased (O’Gara et al., 2000). A study from the same laboratory reported that a mixture of diallyl sulfides (DASS) and dimethyl trisulfide (DMTS) exhibits activity against six enteric pathogens with DMTS being several folds effective compared to DADS (Ross et al., 2001). The activity of DADS and DATS against antibiotic-sensitive and -resistant isolates of *H. pylori* was confirmed by *in vitro* studies (Liu et al., 2008). *In vitro* studies indicated that DAS, DADS, DATS, and diallyl tetrasulfide (DATTS) were effective against *S. aureus*, where DAS was being least effective and the activity increases with the increase in the number of sulfurs. The activity of DATS and DATTS was comparable to standard antibiotics. It was interesting that MRSA was sensitive to all the DASs that were tested (Tsao S.M. and Yin, 2001). Similar results were reported with 237 clinical isolates of *P. aeruginosa* and *K. pneumoniae* including drug-resistant isolates (Tsao S. and Yin, 2001). The addition of DAS and DADS to meat significantly reduced the growth of aerobes and inhibited the pathogenic bacteria (Yin and Cheng, 2003). In line with the above reports, an *in vitro* study investigated the antibacterial activity of not only DASs but also dimethyl sulfides and dipropyl disulfide. The results indicate that they have moderate antibacterial activity against test bacteria, and this activity improves with an increase in the number of sulfurs (Kim et al., 2004). A later study using disk diffusion assay reported that DADS was effective, whereas dipropyl disulfide was not effective against *S. aureus, E. coli*, and *P. aeruginosa* (Casella et al., 2013). Administration of DAS and DADS to diabetic mice infected with MRSA significantly protected the mice by lowering bacteria load in the kidneys. In addition, inflammatory cytokines, namely, IL-6 and TNF-alpha, and coagulation factors C-reactive protein, fibronectin, and fibrinogen were decreased while anticoagulation factors antithrombin III (AT-III) and protein C were increased by DAS and DADS treatment. Moreover, malondialdehyde was decreased upon DAS and DADS, indicating protection from lipid peroxidation by MRSA infection (Tsao et al., 2007). Ally methyl sulfide (AMS) was shown to retard the growth of pleuropneumoniae causing pig pathogen *Actinobacillus pleuropneumonia* and protected the pigs by reducing the lung lesions by 20% (Becker et al., 2012). DAS exhibited concentration-dependent antibacterial activity against *A. actinomycetemcomitans* with and without heat treatment, indicating that DAS is heat stable (Velliyagounder et al., 2012). *In vitro* studies reported that DAS, DADS, and DATS exhibit activity against *C. jejuni* (Lu et al., 2011a; Robyn et al., 2013). The *in vitro* assay treatment of DAS also displays antibacterial activity against *C. sakazakii* and *E. coli* O157:H7 (Lu et al., 2011b; Feng et al., 2014). The enzymatic degradation of alliin, an organosulfur compound that alliinase enzyme converts into allicin and that is further degraded into a variety of organosulfides, resulted in higher percentage of DADS and diethenes showed better antibacterial activity against tested bacteria compared to alkali degradation products of alliin (Wu et al., 2017). Mixtures of DASs with various amounts of mono- to hexasulfides were prepared, and their anti-mycobacterial activity was evaluated. It was found that while all the combinations exhibited some activity, the most potent combination was the one that had higher quantity of DATS (Oosthuizen et al., 2017). Different studies, *in vitro* and *in vivo*, suggest that using GO, combinations, or individual aliphatic disulfides exhibited antibacterial activity against a wide range of microorganisms. It was noticed that the antibacterial activity increases with the increase in the number of sulfur, suggesting that antibacterial activity is mediated by formation of disulfide bonds between the compounds and bacterial protein, mainly enzymes.

## Antibacterial Activity of Allicin

It is an established fact that allicin is an effective, broad-spectrum, and principal antibacterial component of garlic (Ankri and Mirelman, 1999). Allicin was identified as the principal ingredient of garlic that is responsible for the antibacterial activity of a wide variety of bacteria (Cavallito and Bailey, 1944a). Allicin was found to exhibit activity against *M. tuberculosis* including drug-resistant strains (Rao et al., 1946a; Delaha and Garagusi, 1985; Ratnakar and Murthy, 1995). Vancomycin-sensitive and -resistant clinical isolates and standard strains of *Enterococci* species were sensitive to allicin (Jonkers et al., 1999a). Allicin exhibited the best anti-*H. pylori* activity against three strains compared to DASs (O’Gara et al., 2000). A meta-analysis of clinical data indicated that adding allicin to conventional therapy improves the eradication of *H. pylori* infections (Si et al., 2019). Allicin along with related thoisulfinates, allyl methyl, and methyl allyl thiosulfinate were found and purified from acetone garlic extract. Allicin along with allyl methyl and methyl allyl mixture exhibited activity against *H. pylori* and showed synergy when used together (Canizares et al., 2004b). In a recent report, allicin was found to be active against *C. difficile* and other commensal gut bacteria, and no significant synergy was observed when allicin was tested with standard antibiotics (Roshan et al., 2017). The same group reported that allicin did not affect spore germination, but significantly inhibited spore outgrowth of *C. difficile* spores (Roshan et al., 2017). *In vitro* assay found that allicin was effective against 30 strains of *Staphylococcus epidermidis* including methicillin-resistant strains (Perez-Giraldo et al., 2003). A stable aqueous extract of allicin was found to be effective against 30 clinical MRSA isolates, some of which were mupirocin resistant. Aqueous cream of allicin also exhibited activity against tested MRSA strains (Cutler and Wilson, 2004). Similarly, aqueous allicin extract and cream demonstrated anti-Lancefield group B *streptococci* clinical isolate using *in vitro* assays (Cutler et al., 2009). A comparative *in vitro* study of antibacterial activity against *S. aureus* and *E. coli* activity of FGE, allicin, and clinically used antibiotics was performed. The results of the study indicated that fresh garlic was more potent against *S. aureus* compared to allicin and not much difference in activity was noticed against *E. coli* while both bacteria were more sensitive to antibiotics than garlic extract or allicin (Fujisawa et al., 2009). The administration of allicin to rainbow trout through its diet almost eliminated mortality when infected with *Aeromonas hydrophila*, a fish pathogen. In addition, *in vitro* studies also indicated that this bacterium was sensitive to allicin (Nya et al., 2010). It was found that *A. actinomycetemcomitans* was sensitive to allicin, and this activity disappeared upon heating, indicating that allicin is thermolabile (Velliyagounder et al., 2012). Although an *in vitro* assay found that *C. jejuni* was sensitive to allicin, *in vivo* studies indicated that allicin had no significant effect on colonization of *C. jejuni* in broilers. The possible explanation for this could be that the presence of mucus inhibited the activity of allicin *in vitro* (Robyn et al., 2013). In addition to AGE, allicin also exhibits dose-dependent antibacterial activity against Bcc (Wallock-Richards et al., 2014). In an interesting study, allicin vapors were able to exhibit bactericidal activity against MDR lung pathogenic bacteria such as *P. aeruginosa* and *Streptococcus pyogenes* (Reiter et al., 2017). It was found that the active ingredient of Bald’s eyeslave, an Anglo-Saxon medical remedy made up of garlic, onions, bovine bile, and brass effective against *S. aureus* and *P. aeruginosa*, was allicin (Fuchs et al., 2018). Allicin is the most potent antibacterial organosulfur compound found in garlic. The higher activity is thought to be due to the highly reactive sulfoxide group of allicin. However, the stability and solubility of allicin are the challenges in its clinical use. Animal studies highlight the reduced bioavailability and toxicity associated with allicin administration (Amagase et al., 2001).

## Antibiofilm and Antivirulence Properties of Garlic and Its Organosulfur Compounds

Bacterial biofilms are aggregations of bacterial cells in a matrix of extracellular polymeric substances (EPS) that include proteins, nucleic acids, polysaccharides, and lipids that are secreted by the bacteria. The formation of biofilm is a complex process that involves quorum sensing (QS) signaling. QS is also associated with the expression and release of various virulence factors that play a major role in pathogenesis. The formation of biofilm has been strongly associated with bacterial pathogenesis and antibiotic resistance. Therefore, developing strategies to inhibit biofilm formation has been a major area of research for many years. In addition to using synthetic antibiofilm agents, the use of many phytochemicals including garlic and its organosulfur compounds has gained a lot of interest. Table 3 lists the antibiofilm and anti-QS studies that have been performed using garlic and its compounds. AGE was found to inhibit the coagulase activity of *S. aureus* using *in vitro* assays (Fletcher et al., 1974). GO was found to inhibit toxin production by *Clostridium botulinum* type A (Jc et al., 1979). Garlic ointment made by mixing GP with petroleum jelly not only prevented the formation of biofilm but also disrupted the already formed biofilm of bacteria that were isolated from burn wounds (Nidadavolu et al., 2012). DAS was found to kill both planktonic and sessile *C. jejuni* cells in the biofilm much better than ciprofloxacin and erythromycin. FTIR and Raman spectroscopy revealed that DAS treatment altered the proteins and polysaccharides of biofilm and damaged the EPS, which was visualized by electron microscopy (Lu et al., 2012). A genetic screening system was utilized to screen many herbal and pure compounds for their QS inhibition activity, and it was found that garlic exhibited significant inhibition of QS. Microarray transcriptome analysis indicated that the water extract of toluene extract of garlic affected the expression of virulence genes that were controlled by QS. In addition, garlic altered the *in vitro* biofilm to increase the penetration and killing of *P. aeruginosa* in the biofilms by tobramycin (Rasmussen et al., 2005). It was also found that pretreatment of *P. aeruginosa* biofilm to this extract made it more susceptible to tobramycin and polymorphonuclear leukocytes (Bjarnsholt et al., 2005). QS strains were used to identify that the water extract of toluene extract of garlic inhibited LuxR, AhyR, and TarR QS receptors (Bodini et al., 2009). Bioactivity-guided fractionation of garlic extract identified ajoene as quorum sensing inhibition (QSI) and microarray studies revealed that it reduced the expression of few QS-controlled virulence genes of *P. aeruginosa* such as *lasB* and *rhlA*, which increase the production of protease and rhamnolipid, respectively. Similar to previous observations, pretreatment of biofilms with ajoene increased the antibacterial activity of tobramycin on biofilm-associated *P. aeruginosa* (Jakobsen et al., 2012). The QSI activity of ajoene encouraged the screening of a library of compounds to identify a couple of sulfur-containing compounds that were similar to ajoene with QSI activity. Twenty-five disulfide bond-containing compounds were synthesized based on a quantitative structure–activity relationship (QSAR) study. These compounds could reduce the production of virulence factors, which included elastase, rhamnolipid, and pyocyanin. Besides, they were also able to inhibit the infection of *P. aeruginosa* in the murine implant infection model (Fong et al., 2017). The motility and biofilm formation of *P. aeruginosa* was significantly decreased when treated with a combination of ajoene and ciprofloxacin compared to independent treatment with each agent. Ajoene alone and in combination with ciprofloxacin significantly increased the serum sensitivity, phagocytic uptake, and killing of *P. aeruginosa* compared to no treatment. Furthermore, in the *P. aeruginosa* infection-associated murine pyelonephritis model, the combination of ajoene with ciprofloxacin significantly reduced the bacterial load of kidneys and bladder with reduced tissue damage compared to control and individual treatment of ajoene and ciprofloxacin (Vadekeetil et al., 2016). In addition to inhibiting production of long-chain acyl homoserine lactones, ajoene was also found to inhibit *Pseudomonas* quinolone signal (PQS) (Vadekeetil et al., 2015).

**TABLE 3:** Antibiofilm, antitoxin, and anti-QS activity of garlic and its compounds.

Source	Effect	References
AGE	Inhibits coagulase of *S. aureus*	Fletcher et al., 1974
Garlic oil	Inhibits production of toxin by *C. botulinum*	Jc et al., 1979
Garlic ointment	Inhibits formation of biofilm formed by bacterial cells	Nidadavolu et al., 2012
DAS	Inhibits EPS formation in biofilm of *C. jejuni* cells	Lu et al., 2012
Water and toluene extract of Garlic	Inhibits biofilm formed by *P. aeruginosa*	Rasmussen et al., 2005
Garlic extract	Inhibits biofilm and QS complex in *P. aeruginosa*	Bjarnsholt et al., 2005
Garlic extract	Inhibits QS receptors in bacterial cell	Bodini et al., 2009
Ajoene as QSI	Inhibits biofilm formed by *P. aeruginosa*	Jakobsen et al., 2012
Ajoene and 25 disulfide bond-containing compounds	Reduces QS caused infection by *P. aeruginosa*	Fong et al., 2017
Ajoene in combination with Ciprofloxacin	Reduce biofilm related diseases caused by *P. aeruginosa*	Vadekeetil et al., 2016
Allicin	Reduce EPS and virulence factor of *P. aeruginosa*	Lihua et al., 2013
Ajoene	Inhibits *Pseudomonas* quinolone signal	Vadekeetil et al., 2015
DADS	Reduce biofilm related QS and virulent gene of *P. aeruginosa*	Li et al., 2018a
DADS	Inhibits QS, virulent factors, motility, and chemotaxis of *P. aeruginosa*	Li et al., 2018b
Allicin and AGE	Inhibits group A streptococci cytolytic toxin and streptolysin O	Arzanlou and Bohlooli, 2010
DMS	Inhibits downregulation of HilA gene present in *Salmonella* invasion	Antunes et al., 2010
Allicin	Inhibits protease activity of SepB	Arzanlou, 2016
Allicin	Reduction in production of Alpha toxin of MRSA and MSSA	Leng et al., 2011
Allicin and vancomycin	Reduction biofilm formed by *S. epidermidis*	Zhai et al., 2014
Allicin	Reduce thickness of biofilm formed by *S. epidermidis* and down regulate the gene expression	Wu X. et al., 2015
Allicin	Inhibits biofilm formed by *S. aureus*	Perez-Kohler et al., 2015a
Allicin and chlorhexidine	Inhibits biofilm formation by *S. aureus*-infected rabbit hernia model	Perez-Kohler et al., 2015b
FGE	Inhibits biofilm formation of clinical isolates	Farrag et al., 2019

Allicin was also found to not only reduce biofilm formation of *P. aeruginosa* by reducing attachment and EPS production but also reduce the production of virulence factors such as exotoxin A, elastase, pyoverdine, and rhamnolipid (Lihua et al., 2013). Recently, DADS was found to decrease *in vitro* biofilm formation and swarming motility of *P. aeruginosa*. Relative gene expression studies indicated that it reduced the expression of many important QS and virulent genes (Li et al., 2018a). An interesting follow-up study performed an RNA transcriptome and proteome analysis on *P. aeruginosa* upon DADS treatment. The result indicated that all the three QS systems and virulent factors were downregulated by DADS treatment. Also, DADS treatment inhibited systems involved in motility and chemotaxis of *P. aeruginosa* (Li et al., 2018b). *In vitro* studies found that allicin and aqueous garlic (fresh and aged) extract inhibited production of streptolysin O, a cytolytic toxin by all strains of group A streptococci (GAS) (Arzanlou and Bohlooli, 2010). The transcription regulator HilA plays a crucial role in regulating the complex mechanism of *Salmonella* invasion, and it was found that dimethyl sulfide (DMS) downregulates the expression of the *hilA* gene and multiple virulent genes (Antunes et al., 2010). Another toxin produced by GAS is streptococcal pyrogenic exotoxin B (SpeB). The protease activity of SpeB was inhibited by allicin *in vitro*, and it is due to inhibition of truncation of SpeBm, the precursor protein of SpeB (Arzanlou, 2016). The reduction in the production of alpha-toxin by methicillin-susceptible and -resistant *S. aureus* upon treatment with allicin was confirmed by hemolysis and Western blot analysis. In addition, *hla* and *agrA* genes that regulate the production of alpha-toxin were downregulated by allicin (Leng et al., 2011). Administration of allicin alone or with vancomycin significantly reduced biofilm formation by *S. epidermidis* compared to vancomycin or saline treatment in a rabbit prosthetic joint infection model (Zhai et al., 2014). *In vitro* studies indicate that allicin exhibits antibiofilm property against *S. epidermidis*; however, this activity was less compared to water or ethanolic garlic extract. Allicin, water, and ethanolic extract of garlic exhibited antibacterial activity on biofilm-associated bacteria. Allicin decreased the thickness of the biofilm in a concentration-dependent manner. Gene expression studies indicated that allicin treatment of biofilm-associated bacteria resulted in downregulation of *app* and *icaA* genes that are associated with bacterial adhesion whereas only *icaA* was downregulated in planktonic cells (Wu X. et al., 2015). *In vitro* biofilm formation of *S. aureus* on reticular polypropylene mesh used in hernia was partially inhibited by allicin, but this effect diminished over time and a combination of allicin with chlorhexidine had no synergistic effect on the activity. However, allicin and chlorhexidine were cytotoxic individually, but the cytotoxicity was significantly reduced when cells were treated with the combination of both (Perez-Kohler et al., 2015a). To evaluate the effect of presoaking polypropylene mesh in allicin with chlorhexidine on biofilm formation *in vivo*, rabbit hernia model was infected with *S. aureus*, and it was found that combining allicin resulted in lower bacterial clearance and formation of biofilm compared to chlorhexidine treatment (Perez-Kohler et al., 2015b). The spectrum of antibiofilm property of FGE was tested against strong biofilm forming MDR clinical isolates of *P. aeruginosa, K. pneumoniae*, *S. marcescens*, and MRSA using *in vitro* and *in vivo* assays. *In vitro* assay indicated that FGE not only inhibited the formation of biofilm but also eradicated biofilms by these isolates on various surfaces. *In vivo* mice infection studies with *P. aeruginosa* and MRSA studies indicated that FGE significantly improved the survival of the animals and bacteria were not detected in different organs compared to control (Farrag et al., 2019).

The major organosulfur compounds of garlic, namely, allicin, ajoene, and aliphatic sulfides, pose QSI and antibiofilm activity. These activities are most explored in *P. aeruginosa* compared to other bacteria. It is evident from the data that garlic compounds downregulate QS and biofilm-associated genes. However, the precise mechanism in terms of whether the compounds bind and modify the transcription factor or interact with promoters of these genes is yet to be investigated. *In vivo* studies are encouraging to test the use of these compounds in clinical testing.

## Synergistic Effect of Garlic and Its Compounds

The combination therapy sometimes leads to a synergistic effect, which effectively lowers the dose of individual drugs. Similarly, synergistic antibacterial effects were noticed when garlic and its compounds were used in combination with other phytochemicals and antibiotics. Both garlic crude extract and pure allicin exhibited strong synergy with vancomycin against 11 VRE clinical isolates with bacteriostatic action (Jonkers et al., 1999a). Raw garlic extract and commercial garlic tablets displayed synergistic effects against *H. pylori* when used along with omeprazole whereas no such effect was noticed when used in combination with amoxicillin, clarithromycin, and metronidazole (Jonkers et al., 1999b). In a clinical study, administration of allicin along with standard treatment (lansoprazole, clarithromycin, and amoxicillin) improved the percentage of *H. pylori* eradication by 23% in patients (Kockar et al., 2001). The combination of DATS and DATTS was either additive or synergistic when tested in combination with ceftazidime, gentamicin, imipenem, and meropenem except for DAT when used in combination with ceftazidime and gentamicin against ceftazidime and gentamicin-resistant *K. pneumoniae*, respectively (Tsao S. and Yin, 2001). Gentamicin administration induces nephrotoxicity and few reports have indicated that co-administration of aged garlic extract, garlic, S-allyl cysteine, DAS, and DADS ameliorates this nephrotoxicity. It was demonstrated that none of these agents decreased the activity of gentamicin; moreover, SAC, DAS, and DADS have enhanced the antibacterial activity of gentamicin, which makes them safe to use along with gentamicin to protect from nephrotoxicity (Maldonado et al., 2005). The MIC_90_ (concentration of the drug at which 90% of the growth is inhibited) against *S. aureus* of cefazolin and oxacillin were reduced by 128- and 64-fold, respectively, in the presence of 1/4 MIC_90_ of allicin. In the case of *Staphylococcus epidermidis*, MIC_90_ of cefazolin and oxacillin were reduced by 4- and 32-fold, respectively, in the presence of 1/4 and 1/8 MIC_90_ of allicin, respectively. The MIC_90_ of cefoperazone decreased by 16- and 8-fold in the presence of 1/2 and 1/4 MIC_90_ of allicin, respectively, against the cefoperazone-sensitive and -resistant strain of *P. aeruginosa*. The results indicate that allicin in combination with beta-lactam antibiotics results in synergy (Cai et al., 2007). The synergy of allicin with cefoperazone was confirmed against *P. aeruginosa* using the kill curve assay. In the case of tobramycin, certain synergy was observed against *P. aeruginosa* when used in combination with allicin, whereas no synergy was observed in the case of ciprofloxacin (Cai et al., 2008). It is reported that serum of patients that were administered garlic extract with standard antituberculosis had increased antitubercular activity compared to the control group, suggesting a synergistic effect when garlic is given in combination with antituberculosis drugs (Gupta et al., 1999). In the *P. aeruginosa* foreign body infection mouse model, it was noticed that in short-term infection, the bacterial clearance with the combination of ajoene and tobramycin was significantly better compared to placebo or individual treatments. The clearance of bacteria with ajoene alone was also significantly better compared to placebo. In case of long-term infection, the clearing of bacterial cells was significantly improved with ajoene and tobramycin treatment compared to control. However, there was no advantage of combining ajoene to tobramycin in clearing the bacteria (Christensen et al., 2012). Disk diffusion assay results indicated that the crude extract of garlic showed a synergistic effect when used in combination with gentamicin against *E. coli*, and it is interesting to find out that it was the only one out of the tested herbs that did not show any antagonistic effects with any test antibiotics (Ushimaru et al., 2012). In a similar study, antibiotic-resistant *P. aeruginosa* showed sensitivity to cefotaxime and ceftriaxone when FGE was added; this activity was better than FGE alone (Li et al., 2015). Another study reported that the ethyl acetate extract of garlic was antagonistic to the activity of chloramphenicol (Mahomoodally et al., 2018).

MDR *E. coli* isolated from drinking water in Bangladesh were not susceptible to AGE but were found to be sensitive to a combination of 1:1:1 combination of lime juice, garlic, and ginger extract (Rahman et al., 2011). The zone of inhibitions of Tazma honey and garlic crude extract combination were higher than when these were used individually against common pathogenic bacteria (Andualem, 2013). Garlic essential oil in combination with essential oils from several other species did not result in any synergy against tested pathogenic bacteria (Bag and Chattopadhyay, 2015). The *in vitro* interaction studies of a combination of AGE and Manuka honey against extended-spectrum beta-lactamase-producing *E. coli* showed different effects on different isolates ranging from synergy, additive, and indifferent to antagonistic effects (Idris and Afegbua, 2017). Mechanistic studies to understand the synergistic effects of garlic with antibiotics or other chemicals are lacking. Such studies are required for encouraging the use of garlic compounds to complement conventional medicine.

## Mechanisms of Antibacterial Activity of Garlic Organosulfur Compounds

The principal active ingredient responsible for the antibacterial activity of garlic was identified to be allicin (Cavallito and Bailey, 1944a). This finding was immediately followed by the observation that cysteine and other sulfhydryl-containing compounds inhibited the antibacterial activity of allicin, leading to the hypothesis that allicin might exert its antibacterial effect by reacting with sulfhydryl groups of the bacterial proteins (Cavallito and Bailey, 1944b; Cavallito et al., 1945). Allicin would react to sulfhydryl groups of cysteines irreversibly and will not be available to react with the sulfhydryl groups of the enzymes. The ability of allicin to inhibit various sulfhydryl enzymes indicates that the mechanism of antibacterial action of allicin is by reacting with the sulfhydryl groups of the many metabolically important bacterial enzymes (Wills, 1956). The action of allicin is mostly non-specific as it is found to inhibit urease, papain, amylase, and alcohol dehydrogenase. NMR experiments identified *S*-allylmercaptocysteine, confirming the reaction between allicin and sulfhydryl group of cysteine (Rabinkov et al., 1998). The rapid permeability of allicin through lipid bilayers supports the idea of allicin able to reach and react with sulfhydryl groups of bacterial proteins (Miron et al., 2000). The treatment with reducing agents such as β-mercaptoethanol and dithiothreitol resulted in the loss of allicin’s antibacterial activity, indicating that allicin forms disulfide bonds with the sulfhydryl groups of enzymes (Rabinkov et al., 1998; Jonkers et al., 1999a). Mass spectrometry and Raman spectrum analysis confirm that allicin enters the cell rapidly and reacts with cysteine and glutathione sulfhydryl groups (Fujisawa et al., 2009; Miron et al., 2010; Lu et al., 2011a). The enzymatic activity of bacterioferritin comigratory protein (BCP) from *B. cepacia*, which has two catalytically cysteines, was inhibited by allicin and mass spectrometry analysis confirmed that S-allyl thiol groups were added to these cysteines by allicin (Wallock-Richards et al., 2014). The inhibition of trypsin-like protease and general protease activity of *P. gingivalis* cell extract by AGE suggests that the antibacterial activity of garlic could be due to inhibition of proteolysis (Bakri and Douglas, 2005). Streptolysin O and mature Streptococcal pyrogenic exotoxin B of *Streptococci* that contain functionally important cysteines were inhibited by allicin and the addition of DTT reversed the inhibition (Arzanlou and Bohlooli, 2010; Arzanlou, 2016). Mass spectrometric proteomic analysis of cytoplasm of *E. coli* treated with allicin revealed 73 S-thioallylated proteins including some essential metabolic enzymes. It was shown that allicin reacts with low-molecular-weight cellular thiols such as glutathione (GSH) causing oxidative stress (Muller et al., 2016). Overall, these studies establish that the mechanism of antibacterial activity of allicin is by reacting with the sulfhydryl attaching allythio group through a disulfide bond. However, it should be noted that the mechanism of action of allicin is non-specific, which could make it cytotoxic. Alternatively, it has been reported that treatment of allicin inhibits DNA, RNA, and protein synthesis in bacteria, and the inhibition of RNA synthesis is more profound (Feldberg et al., 1988). *In vivo* study revealed that allicin modulates immunological parameters such as increased phagocytic and serum lysozyme activity to protect rainbow trout fish from *A. hydrophila* infection (Nya et al., 2010).

Allicin is highly unstable and is degraded into various organosulfide compounds (Figure 1B). The organosulfides also have been reported to show activity against a wide range of bacteria. Different studies have shown that organosulfides constitute a majority of GO. The mechanism of action of organosulfides, like allicin, is to react with free sulfhydryl groups of enzymes. However, organosulfides are not as reactive as allicin due to the absence of oxygen that is bound to sulfur in allicin. The activity of *H. pylori* arylamine *N*-acetyltransferase was inhibited in the presence of DAS and DADS, suggesting that these compounds exert antibacterial activity by inhibiting bacterial enzymes (Chung et al., 1998). In a more recent report, DAS was also found to inhibit the activity of the GST enzyme (Velliyagounder et al., 2012). Like allicin, addition of cysteine has reduced the antibacterial activity of GO against *E. aerogenes*, suggesting that sulfides also react with free sulfhydryl groups of enzymes (Ross et al., 2001). A recent study established that the antibacterial activity of diallyl polysulfides is due to their ability to react with sulfhydryl groups of various enzymes of *B. subtilis* such as bacillithiol and CoA and with amino acid cysteine (Kyo et al., 2001).

FTIR and Raman spectroscopy analysis revealed that treatment of *C. jejuni*, *C. sakazakii*, *E. coli* O157:H7, *L. monocytogenes*, and *Bifidobacterium* species with garlic crude extract and allyl sulfides causes many spectral changes that indicate the interaction of these agents with various cellular components; most notable changes indicate reaction with sulfhydryl groups, modification of cell membrane, and wall components to damage and destroy the integrity of the cell. These observations were consistent with the electron microscopy data that showed damage to cell wall and membrane (Lu et al., 2011a, b, 2012; Booyens et al., 2014; Booyens and Thantsha, 2014; Feng et al., 2014). Treatment of *C. jejuni* with DAS decreased the cellular ATP levels and increased the level of cellular protein in the culture, suggesting loss of cell membrane integrity (Lu et al., 2012).

A global proteomic analysis was performed to determine the mechanism of the anti-*H. pylori* effect of DATS. The results of 2D gel electrophoresis of proteins showed that upon treatment with DATS, proteins involved in metabolism, biosynthesis, bacterial virulence, and redox reactions were downregulated while stress response chaperon proteins were upregulated. The production of CagA and VacA virulent protein was decreased due to DATS treatment (Zare et al., 2008). A study performed RNA sequencing to study the changes in the global transcriptome of *C. sakazakii* upon treatment with DAS. Although there were a large number of genes that were up- and downregulated by the DAS treatment, clusters of genes that are related to cell shape and wall maintenance and lipopolysaccharide synthesis were upregulated while RNA and amino acid biosynthetic genes were downregulated. This indicates that DAS causes injury to the cell wall and membrane and decreases general metabolism (Feng et al., 2014).

The anti-*C. sakazakii* activity of ajoene was also diminished by the addition of cysteines, indicating that its antibacterial activity also involves reacting with sulfhydryl groups of bacterial enzymes. The transcriptome analysis of *C. sakazakii* treated with ajoene showed that the NADH expression factor and nitrate reductase gene were downregulated, which is related to reactive oxygen species reactions. In addition, flagellum and bacterial motility genes were downregulated, suggesting that ajoene negatively impacts biofilm formation (Feng et al., 2014).

**FIGURE 2:**
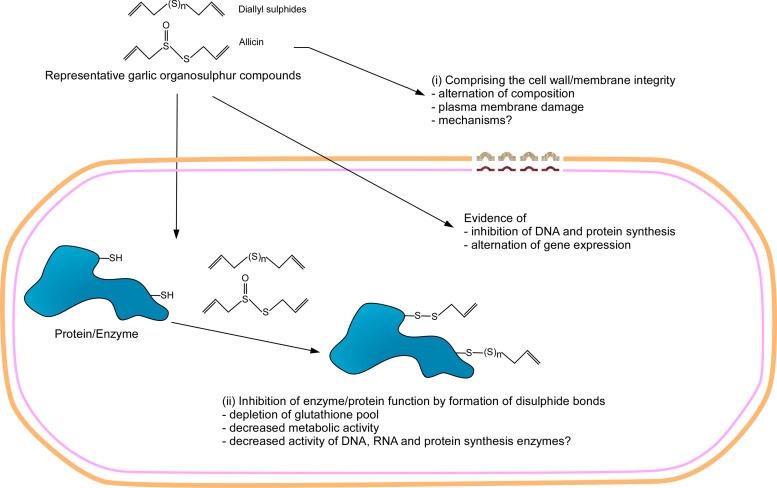
Illustration of the mechanism of action of garlic organosulfur compounds. Garlic organosulfur compounds exert their antibacterial activity mainly through two mechanisms: (i) The organosulfur compounds are highly reactive with sulfur having the capability to form disulfide bonds with the free sulfhydryl groups of proteins including enzymes. The formation of disulfide bonds renders the enzyme inactive, resulting in the death of bacteria. (ii) The organosulfur compound interacts with the cell membrane of bacteria. This interaction compromises the integrity of the cell membranes of the bacteria leading to leakage of cell content leading to death. In addition, it is also thought that garlic organosulfur compounds interfere with protein production, DNA replication, and alter gene expression.

In summary, two main mechanisms of action of garlic organosulfur compounds emerged from the reported studies: (1) the reaction of garlic compounds to the free sulfhydryl group on the proteins and/or enzymes to inactivate them, and (2) the disruption of composition and integrity of bacterial cell membrane and/or cell wall. Besides, some work also suggests that garlic compounds could also have a global effect on DNA, RNA, and protein synthesis. These mechanisms are observed in both Gram-positive and Gram-negative bacteria, suggesting that garlic and its compounds use similar antibacterial mechanisms for both groups of bacteria. However, the activity of the compounds is not specific, which could restrict their clinical application. The mechanism of action of garlic and its compound has been summarized in Figure 2.

## Emerging Novel Techniques and Opportunities to Use Garlic as Novel Antibacterial Agent

The emergence of various technologies such as nanotechnology, refined organic synthesis methods, and specialized drug delivery methods provide ample opportunities to use garlic organosulfur compounds as novel antibacterial agents. Green nanoparticle synthesis in recent times has emerged as a powerful tool to use phytochemicals to not only synthesize nanoparticles but also improve the antibacterial functions of these chemicals and particles (Wang and Vermerris, 2016).

Garlic extract has been used for the green synthesis and stabilization of silver nanoparticles. Eco-friendly garlic-silver nanoparticles synthesized using garlic clove extract displayed a greater antibacterial and antibiofilm activity on clinically important pathogens such as MRSA and *P. aeruginosa* compared to garlic extract or silver nitrate (Vijayakumar et al., 2019). A novel highly active molecule, (2E, 2E)-4,4-trisulfanediylbis (but-2-enoic acid) (TSDB) was synthesized through comparative molecular field analysis (COMFA) using the structure of DATS. TSDB displayed a robust inhibitory effect against *S. aureus* at low concentration. TSDB treatment increased the conductivity better than DATS, indicating better membrane penetration. The increase in the levels of protein and no change in the levels of alkaline phosphate in culture upon treatment with TSDB and DATS compared to control suggest damage to the cell membrane but not so much to the cell wall (Wu et al., 2018). Solgel prepared using tetraethyl orthosilicate was loaded with 20% ethanol extract of garlic, which displayed controlled release and stability of garlic components with increased antibacterial and antibiofilm activity against MRSA (Girish et al., 2019). GO microspheres were monodispersed in water by microemulsion technique to overcome its volatile characteristics and poor aqueous solubility. The study specified that the water-dilutable microemulsion that is formed by GO encapsulated in a nanoparticle vector is effective in preventing *S. aureus* than *E. coli* (Zheng et al., 2013). In another study, wild garlic (*Allium ursinum* L.) extract was encapsulated using spray congealing technology to shield its valued active compounds and expand its oral bioavailability. The encapsulation led to an enhancement of the extract dissolution performance as well as an improvement in the solubility of more than 18-fold compared to the pure extract. Microparticles were stable over a 3-month period, showing only a minor decrease in the content of active compounds (allicin and *S*-methyl methane thiosulfonate) and upholding a good antimicrobial activity. The study suggests that such spray congealing technology can be used to improve the solubility, bioavailability, and stability of the garlic active ingredients including allicin without affecting their antibacterial properties (Tomsik et al., 2019). The stability of phytochemicals present in GO, mainly allicin, was improved when biogenic nanoscale mesoporous silicon derived from the silicon-accumulator plant Tabasheer (*Bambuseae*) was used as a potential carrier as the antibacterial activity of this material was better than GO only control (Le et al., 2017). More research should be focused to make similar nanoparticles, emulsions, and novel formulations using pure garlic compounds, mainly allicin, to improve their stability. *In vitro* studies with allicin aerosol and vapors using a lung model demonstrated the antibacterial efficacy of allicin with a correlation between aerosol deposition pattern and bacterial growth inhibition. Interesting synergy was observed with allicin that was administered with ethanol against *E. coli* (Reiter et al., 2020). It was interesting to note that DAS is not a strong antibacterial compound, but when given in combination with zinc oxide nanorods as an emulsion, it displayed a synergistic effect against *S. aureus* and MRSA bacteria under *in vitro* and *in vivo* conditions (Rauf et al., 2018). In a study, SAC, which is not antibacterial by itself, exhibited antibacterial activity when in complex with palladium (II) against *E. coli*, *P. aeruginosa*, and *S. aureus* (Spera et al., 2011). Organosulfides were converted into nano-iron sulfides with 500-fold superior antibacterial activity against pathogenic and drug-resistant bacteria compared to compounds themselves. The nano-iron sulfides released hydrogen polysulfanes and topical application in animal models resulted in reduced biofilm formation and accelerated wound healing (Xu et al., 2018).

Leontiev et al. (2018) developed a series of allicin analogs and evaluated their antimicrobial properties and thermal stability against bacteria and the model fungus *Saccharomyces cerevisiae*. Here, dimethyl-, diethyl-, diallyl-(allicin), dipropyl-, and dibenzyl-thiosulfinates form a series of molecules with increasing molecular mass and hydrophobicity, which would be anticipated to affect physical characteristics such as rate of diffusion, volatility, and membrane permeability, all of which are expected to affect the antibacterial properties of the molecules. In this study, the more volatile compounds showed noteworthy antimicrobial properties *via* the gas phase. Thiosulfinates differed in their effectivity against specific organisms, and some were thermally more stable than allicin. These results encourage the application of garlic-based compounds in medicine and agriculture either singly or in combination with other antimicrobials (Leontiev et al., 2018). Attaching *N*-propylthiol (similar chemistry to allicin) to ciprofloxacin increased the sensitivity of MRSA toward ciprofloxacin, suggesting that combination chemistry with garlic organosulfur could potentiate existing antibiotics (Sheppard and Long, 2016). Another study screened a chemical library composed of 19 synthesized pyridyl disulfides that emulate the chemical reactivity of allicin for antimicrobial activity against Gram-positive species including VRSA. The study identified pyridyl disulfides as stable alternatives to allicin with a similar narrow-spectrum profile and are thought to function as pro-oxidants like that of allicin (Sheppard et al., 2018).

As garlic is consumed regularly all over the world, it is considered non-toxic without any side effects. However, there are limited reports of toxic side effects of garlic and its constituents. In some individuals, contact with garlic and its constituents (especially oil-soluble sulfur compounds) leads to skin irritation and dermatitis (Jappe et al., 1999). *In vivo* studies administering garlic juice resulted in stomach damage. Garlic juice rich in allicin and allicin itself cause damage to the intestinal epithelial mucosa (Kodera, 1997). Allicin was also reported to immobilize sperms *in vitro* (Qian et al., 1986). *In vitro* cytotoxicity studies showed that DAS did not affect the cell growth or viability, whereas both DAS and allicin changed the morphology of cells. Allicin also significantly decreased the metabolic activity of cells (Velliyagounder et al., 2012; Perez-Kohler et al., 2015a). The concentration at which these effects are noticed are relatively high and more studies need to be done to evaluate the toxicity of garlic and its compounds at concentrations that exhibit antibacterial effects.

## Conclusion

**FIGURE 3:**
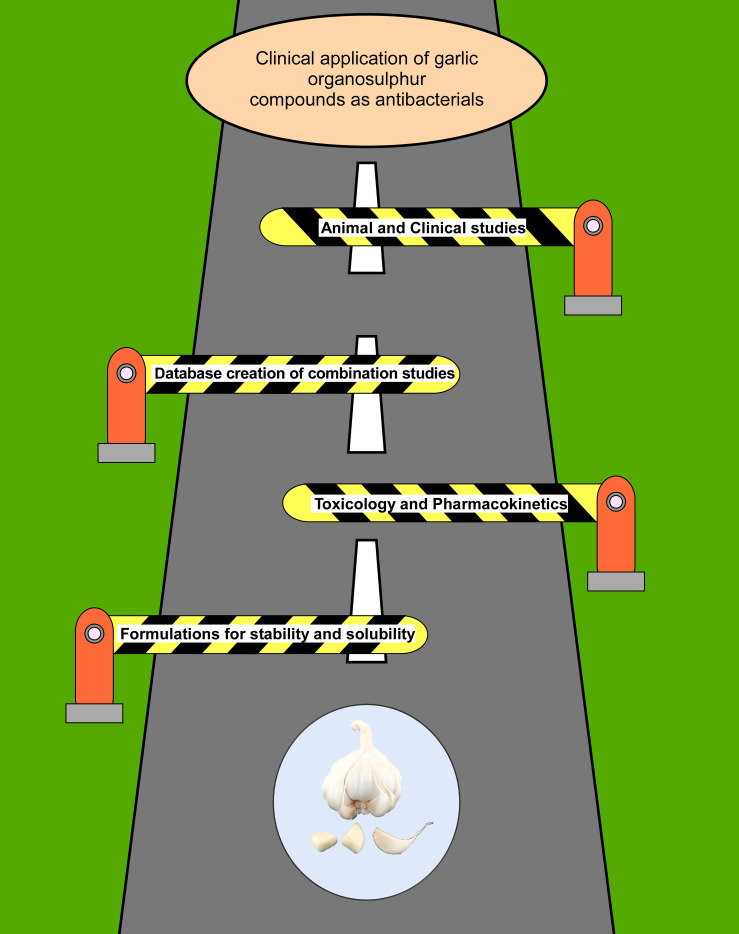
Cartoon representation of the challenges that should be crossed for garlic/garlic compounds to be used in the clinical setting as antibacterials. The road to developing garlic/garlic compounds into novel clinically relevant antibiotics is subject to crossing the barrier shown in the cartoon, which are as follows: (1) the development of new formulations that would improve the specificity, stability, and solubility of garlic organosulfur compounds; (2) to access the safety and bioavailability of organosulfur compounds by performing toxicology and pharmacokinetic studies with various compounds administered through different routes; (3) to avoid repetition and encourage *in vivo* studies, creation of a database to record the *in vivo* and *in vitro* antibacterial studies of garlic compounds alone and along with other antibiotics or phytochemicals; and (4) more pre-clinical and clinical studies should be performed to access the safety and efficacy of these compounds. Research in these areas will ensure the use of garlic-based novel antibacterials in the clinical setting.

The extensive research strongly indicates that garlic organosulfur compounds exhibit strong antibacterial activity against a wide range of bacteria including MDR strains. Although garlic organosulfur compounds have been known to be excellent antibacterial compounds, not much progress has been made in the direction of utilizing them clinically to tackle the problem of antibiotic resistance. The toxicity data of garlic and its compounds from animal studies are inconsistent with some studies reporting no toxic effect, whereas some report inflammation and toxic effects. The lower stability, solubility, and bioavailability of these compounds have hindered their use in the clinical setting. However, the organosulfur compounds are attractive because their bactericidal activity is exerted through multiple mechanisms, making it difficult for bacteria to develop resistance. Another concern that should be addressed for the use of garlic compounds is their toxicity and specificity to use them as antibacterial agents. Although a great deal of research has been done on the antibacterial potential of garlic and its compounds, there are recent gaps that need to be filled to utilize them as antibacterial agents in clinical settings. The first major area where more research should be focused is to develop robust and economical extraction or synthesis procedures that would yield pure garlic compounds. Besides, most of the organosulfur compounds of garlic are not water soluble and are unstable. Thus, formulations using advanced nanoparticle or emulsion techniques should be developed with improved solubility and self-life. A database should be created to curate the antibacterial data of garlic compounds by themselves and in combination with other antibiotics, which should be performed under pre-established guidelines. Such a database can be used by artificial intelligence tools to predict the most effective combinations for testing or treatment. Most of the research reports the *in vitro* antibacterial activity of garlic compounds that are important but not necessarily translate to *in vivo* conditions. Therefore, future research should be focused on validating the antibacterial activity of garlic compounds in animal infection models. Furthermore, elaborate toxicology and pharmacokinetic studies with respect to different pure organosulfur compounds, administered amount, and route should be performed. Clinical studies of garlic compounds except for anti-*H. pylori* activity has not been performed. As garlic is approved by FDA as “generally recognized as safe,” garlic products should be administered in addition to standard antibiotics in the clinical setting to establish the role of garlic in complementary medicine. All these gaps in the research have been summarized in a cartoon (Figure 3). Recent advances in science and technology such as combinatorial and refined chemical synthesis techniques, nanotechnology, bioinformatics, computation tools, and advanced formulation can effectively overcome the challenges to develop the garlic organosulfur-based novel antibiotics. In the light of rapid emergence of antibiotic resistance, it is warranted that more research should be focused to understand the precise mechanism of action at a molecular level of organosulfur compounds. The organosulfur compounds have the potential to make a huge impact on human health by decreasing the mortality associated with bacterial infections.

## Author Contributions

All authors listed have made a substantial, direct and intellectual contribution to the work, and approved it for publication.

## Conflict of Interest

The authors declare that the research was conducted in the absence of any commercial or financial relationships that could be construed as a potential conflict of interest.

## Publisher’s Note

All claims expressed in this article are solely those of the authors and do not necessarily represent those of their affiliated organizations, or those of the publisher, the editors and the reviewers. Any product that may be evaluated in this article, or claim that may be made by its manufacturer, is not guaranteed or endorsed by the publisher.

## Abbreviations

MDR: multi-drug-resistant
FGE: fresh garlic extract
GP: garlic powder
GO: garlic oil
AGE: aqueous garlic extract
QS: quorum sensing
SAC: *S*-allyl cysteine
STEC: Shiga toxin-producing *Escherichia coli*
MRSA: methicillin-resistant *Staphylococcus aureus*
VRSA: vancomycin-resistant *Staphylococcus aureus*
VRE: vancomycin-resistant *enterococci*
DAS: diallyl sulfide
DADS: diallyl disulfide
DATS: diallyl trisulfide
DATTS: diallyl tetrasulfide
MIC: minimum inhibitory concentration
Bcc: *Burkholderia cepacia* complex
EPS: extrapolysaccharide
QSI: quorum sensing inhibition.
